# Increased copy-number and not DNA hypomethylation causes overexpression of the candidate proto-oncogene CYP24A1 in colorectal cancer

**DOI:** 10.1002/ijc.28143

**Published:** 2013-04-05

**Authors:** Julia Höbaus, Doris M Hummel, Ursula Thiem, Irfete S Fetahu, Abhishek Aggarwal, Leonhard Müllauer, Gerwin Heller, Gerda Egger, Ildiko Mesteri, Sabina Baumgartner-Parzer, Enikö Kallay

**Affiliations:** 1 Department of Pathophysiology, Medical University of ViennaVienna, 1090, Austria; 2 Department of Internal Medicine III, Medical University of ViennaVienna, 1090, Austria; 3 Clinical Institute of Pathology, Medical University of ViennaVienna, 1090, Austria; 4 Department of Internal Medicine I, Medical University of ViennaVienna, 1090, Austria; 5 Comprehensive Cancer Center, Medical University of ViennaVienna, 1090, Austria

**Keywords:** CYP24A1, colorectal cancer, 20q13.2, methylation, proliferation

## Abstract

In colorectal cancer (CRC) the vitamin D catabolizing enzyme 1,25-dihydroxyvitamin D 24-hydroxylase (CYP24A1) is overexpressed with a potentially significant, positive impact on the catabolism of 1,25-dihydroxyvitamin D_3_ (1,25-D_3_). However, the underlying mechanism of CYP24A1 overexpression is poorly understood. In the present study, we investigated possible causes including hypomethylation of the CYP24A1 promoter, amplification of the *CYP24A1* gene locus (20q13.2), and altered expression of *CYP24A1*-specific transcription factors. We quantified CYP24A1 gene copy-number, performed bisulfite sequencing of the *CYP24A1* promoter to assess DNA methylation, and measured mRNA expression of CYP24A1, 25-hydroxyvitamin D 1α-hydroxylase (CYP27B1), vitamin D receptor (VDR) and retinoid X receptor (RXR). We found that 77 (60%) out of 127 colorectal tumors showed increased CYP24A1 gene copy-number and that more than 6 copies of *CYP24A1* correlated positively with CYP24A1 mRNA expression suggestive of a causal relationship. No differences in *CYP24A1* promoter methylation were found between tumor tissue and adjacent mucosa from the same patient or between tissues with high or low mRNA expression, thus excluding DNA hypomethylation as a possible cause of CYP24A1 overexpression in CRC. Furthermore, mRNA expression of several factors involved in replication licensing positively correlated with CYP24A1 mRNA expression, raising the possibility that CYP24A1 overexpression might favor increased proliferation in tumors by suppressing local 1,25-D_3_ levels. We conclude that high copy-number gain is a key determinant of CYP24A1 overexpression in CRC. Other postulated causes of CYP24A1 overexpression including promoter hypomethylation and enhanced VDR and/or RXR expression do not appear to be involved.

What’s new?

Recently, it has been suggested that the association between colorectal cancer and reduced levels of circulating vitamin D may be related to overexpression of the vitamin D-catabolizing enzyme, CYP24A1 in the tumor. In this search for a mechanistic explanation, increased *CYP24A1* gene copy number was associated with the enzyme’s overexpression in 60 percent of colorectal tumors, and expression was correlated strongly with proliferation markers. The findings suggest that CYP24A1 overexpression is likely to deplete tumor calcitriol (1,25-dihydroxyvitamin D_3_) levels, possibly increasing the proliferative potential of the tumors.

Colorectal cancer is the third most common cancer in the world with over 1.2 million new cases in 2008. Colorectal cancer risk is inversely associated with the vitamin D status of the patient and epidemiological studies suggest that vitamin D supplementation decreases colorectal cancer incidence.^1^,^2^ 25-hydroxyvitamin D_3_ (25-D_3_, calcidiol) is the circulating form of vitamin D. It is hydroxylated at position C-1 by 25-hydroxyvitamin D_3_ 1α-hydroxylase (CYP27B1) to form the most active vitamin D metabolite 1,25-dihydroxyvitamin D_3_ (1,25-D_3_, calcitriol). Systemic levels of 1,25-D_3_ are controlled by the synthesizing enzyme CYP27B1 and the catabolizing enzyme 1,25-dihydroxyvitamin D 24-hydroxylase (CYP24A1) in the kidneys.^3^ To avoid hypercalcemia induced by excessively high blood levels of 1,25-D_3_, 1,25-D_3_ provides feedback inhibition of CYP27B1 expression and coordinately upregulates the expression of CYP24A1.^4^ The main endocrine function of 1,25-D_3_ is the modulation of calcium and phosphate homeostasis and bone mineralization.^5^

Expression of CYP27B1 and CYP24A1 is not limited to the kidneys, but is found in various tissues including breast and prostate, as well as colon.^6^ Extra-renally produced 1,25-D_3_ acts in an autocrine/paracrine manner^5^ and is considered to be anti-tumorigenic by regulating cell proliferation, apoptosis, angiogenesis and differentiation.^7^,^8^ The 1,25-D_3_ catabolizing enzyme CYP24A1 is overexpressed in several malignancies including tumor samples from patients with colorectal cancer.^9^ This abnormally high expression promotes the catabolism of local 1,25-D_3_ and appears to suppress its anti-tumorigenic effects. Thus, *CYP24A1* has been identified as a proto-oncogene in colorectal and other cancers.^10^–^12^ The mechanisms responsible for upregulation of *CYP24A1* in colorectal carcinogenesis are not well understood. Changes in promoter methylation and gene amplification have been identified as possible causes of aberrant *CYP24A1* expression in cancer.^13^,^14^

DNA methylation is an epigenetic alteration and refers to the addition of a methyl group to cytosine bases in CpG dinucleotides. CpGs can either occur in isolation or in so-called CpG islands (CGIs) that are typically ≥500 bp, with GC contents that exceed 55%. CGIs are found in around 60% of gene promoter regions including the *CYP24A1* promoter (bases −467 to +1273).^15^ During carcinogenesis, global DNA hypomethylation occurs, causing genomic instability and activation of proto-oncogenes.^16^,^17^ The *CYP24A1* promoter contains regulatory elements including specificity protein 1 (SP1) binding sites and two vitamin D responsive elements (VDRE 1+2) controlling both basal and 1,25-D_3_ induced transcription,^18^ while distal enhancer elements^19^ are not located within CGIs. Reporter assays indicated that *CYP24A1* promoter methylation lowers basal transcription and reduces responsiveness of *CYP24A1* to 1,25-D_3_ dependent transcription,^20^,^21^ whereas demethylation promotes transcription.^22^ In prostate cancer, increased promoter methylation coincided with CYP24A1 downregulation.^21^,^23^ Besides osteoblastic ROS cells and prostate cancer cell lines, the *CYP24A1* promoter is methylated also in healthy human placenta.^20^–^22^,^24^ Hypermethylation of other components of the vitamin D system were reported in breast cancer (*VDR* and *CYP27B1*),^25^,^26^ thus indicating an epigenetic control of the vitamin D system. A possible epigenetic control of CYP24A1 has been discussed in various recent reviews but has not been tested in the colon so far.^14^,^27^ Our study is the first to assess the methylation status of the *CYP24A1* promoter in normal colon and colon cancer tissue, testing the hypothesis that the low expression of CYP24A1 in the normal colon mucosa could be due to promoter hypermethylation and that hypomethylation results in CYP24A1 overexpression in tumor tissue.

In addition to changes in methylation status, genomic instability in cancer results in chromosomal rearrangements and gene amplification. Although, copy-number gains may conceivably occur on all chromosomes, in colorectal cancer they are found with the highest frequencies on chromosomes 13q, 8q, and 20q.^28^ These regions are less frequently amplified in adenomas, suggesting that they might provide proliferative and/or metastatic advantage for the tumor and might be involved in the transition from adenoma to adenocarcinoma.^28^,^29^ *CYP24A1* is located on 20q13.2, a region that is amplified in various malignancies including breast and pancreas, as well as colon.^12^,^30^ In previous studies, a broad range of colorectal tumors (9–92%) have been reported to exhibit 20q13 copy-number gain.^31^–^33^

The interest in using vitamin D as a potential cancer preventive factor or even as an adjuvant chemotherapeutic substance has increased tremendously in the last few years. However, the beneficial effects of 1,25-D_3_ are blunted by the upregulation of its degrading enzyme CYP24A1. Therefore, understanding the mechanisms behind CYP24A1 overexpression in tumors is of utmost importance, in order to take advantage of the entire anti-tumorigenic potential of vitamin D.

In this study, we investigated possible mechanisms underlying the upregulation of CYP24A1 in colorectal cancer. The current findings indicate that the DNA methylation state of the *CYP24A1* promoter does not play a role in the pathogenesis of colorectal cancer. We focused on determining *CYP24A1* copy-number and expression levels in the same patient cohort, demonstrating that high copy-number gains are closely associated with increased CYP24A1 mRNA and protein expression. Furthermore, our data suggest that one of the consequences of high CYP24A1 expression in colorectal tumors is increased proliferative potential.

## Material and Methods

### Tissue samples

Tissue samples (fresh frozen) were collected at the General Hospital of Vienna and Rudolfsstiftung Hospital after approval by the ethics committee of the Medical University of Vienna. Formal, written consent was obtained from all patients. 138 colorectal cancer tissue and adjacent mucosa from the same patient were collected, graded, and classified according to the TNM system by a pathologist. Because of the limited quantity of tissue samples we were unable to extract both RNA and DNA from some of the patients.

#### RNA isolation and reverse transcription

RNA was extracted with Trizol (LifeTechnologies (Invitrogen), Vienna, Austria) according to the manufacturer’s instructions and measured with a NanoDrop ND-1000 (Peqlab, Erlangen, Germany). RNA integrity was assessed by ethidium bromide staining on agarose gels and 2 µg of total RNA were reverse transcribed with RevertAid H Minus Reverse Transcriptase (Fermentas, St. Leon-Rot, Germany) using random hexamer primers according to the manufacturer’s instructions. cDNA was diluted 1:8 in water before qRT-PCR.

#### Quantitative reverse transcription PCR

We screened various housekeeping genes and found beta-actin to be stably expressed in colorectal cancer and normal mucosa. Quantitative reverse transcription PCR (qRT-PCR) was performed as described before.^9^ Samples were run in duplicates with POWER SYBR GREEN Mastermix (LifeTechnologies, Applied Biosystems), Vienna, Austria) on a Step One Plus qRT-PCR machine (LifeTechnologies). All values were set relative to total RNA calibrator and ΔΔC_T_ was calculated relative to the housekeeping gene. Primer sequences of CYP24A1, VDR, CYP27B1, β-actin have been described before.^9^ Primer sequences for CYP3A4 (fwd: CAGGAGGAAATTGAGGCAGTTTT, rev: GTCAAGATACTCCATCTGTAGCAGAGT), RXRα (fwd: GGACATGCAGATGGACAAGAC, rev: CCTTGGAGTCAGGGTTAAAGAG), MCM2 (fwd: GCCAAGATGTACAGTGACCTGA, rev: GATGTGCCGCACCGTAAT), MCM4 (fwd: TTGAAGCCATTGATGTGGAA, rev: GGCACTCATCCCCGTAGTAA), MCM7 (fwd: CGGTGCTGGTAGAAGGAGAG, rev: AAACCCTGTACCACCTGTCG), CDC6 (fwd: CCTGTTCTCCTCGTGTAAAAGC, rev: GTGTTGCATAGGTTGTCATCG).

#### Immunofluorescence staining

Sections (5 μm) of paraffin-embedded tissue were incubated for 25 min at 60°C, deparaffinized, and rehydrated. After washing in phosphate-buffered saline (PBS, pH 7.2), we performed antigen-retrieval by boiling sections in 0.05% citraconic anhydride. Sections were washed in PBS, permeabilized in PBS, 0.2% Tween-20 (Sigma-Aldrich, Munich, Germany) for 15 min and blocked with 10% goat serum in PBS (Jackson ImmunoResearch, Suffolk, UK) for 45 min. CYP24A1 primary antibody was diluted 1:1,000 in 10% goat serum. Rabbit IgG (Abcam, Cambridge, UK) was used as negative control. After extensive washing, sections were incubated with Dylight 549 goat-anti-rabbit IgG (1:500, Vector Laboratories, UK). Nuclei were stained with Dapi (Roche, Vienna, Austria) for 10 min and coverslips were mounted using Fluoromount-G (Southern Biotech, Birmingham, AL). Whole tissue slide images were acquired using TissueFAXS 2.04 (TissueGnostics, Vienna, Austria). Staining intensities were classified as weak staining (*i*), weak positive (*ii*), moderate positive (*iii*), strong positive (*iv*) and highly positive (*v*).

#### DNA isolation and copy-number assay

DNA was isolated by standard proteinase K (Sigma-Aldrich) digestion and subsequent phenol/chloroform extraction. DNA copy-number of *CYP24A1* (fwd: GCTAACATCATATCCAACTCAG, rev: TGAAGTGTAAACCAGCAGTG) and *RNaseP* (fwd: CAGCGAAGTGAGTTCAATGG, rev: GGAGGAGAGTAGTCTGAATTGG) was assessed in duplicates by POWER SYBR GREEN (LifeTechnologies) qPCR on Step One Plus Real Time Machine (LifeTechnologies). The assay was adapted for *CYP24A1* (20q13.2) and *Ribonuclease P RNA component H1* (14q11.2) after the method of Ponchel *et al*.^34^ Primers were used at a final concentration of 500 nM (*CYP24A1*) and 300 nM (*RNAseP*) to achieve a ratio of 1:1 for both genes in samples with no gene amplifications. For this, ΔCt was calculated to achieve a value of 0 by the formula: ΔC_T_ = (C_T_ of the target *CYP24A1*) − (C_T_ of the reference *RNAseP*). Human placenta was used as calibrator and the assay was validated with normal human colon and kidney as controls for normal copy-number. The melanoma cell line VM-17 was used as a positive control for gene amplification of *CYP24A1* but not *RNaseP*, as previously determined by CGH.^35^,^36^ ΔΔC_T_ was calculated, values below 2 were considered as normal copy-number (less than four copies), values above 2 as increased copy-number gain (more than four copies). For assay validation, we performed Fluorescence In Situ Hybridization (FISH) as described before (data not shown).”^37^

#### Bisulfite genomic sequencing

Bisulfite Genomic Sequencing Primers were designed using Methyl Primer Express v1.0 (LifeTechnologies). Region 1 contains 45 CpGs and represents the proximal promoter region (−494 to +3, primers fwd: ATTTTAGTTTAGGTTGGGGGTATTT, rev: CCATATTCCTATACCCAAAAACCAT), Region 2 is localized at the transcription start site and contains 39 CpGs (−18 to +609, primers fwd: TTTTTGGGTATAGGAATATGGAGAG, rev: CCCAACAATAACCAACTAATAAAAC). DNA was bisulfite-converted with the EpiTect Bisulfite Kit (Qiagen, Hilden, Germany). PCR amplification was performed using HotStarTaq DNA Polymerase (Qiagen), run on a 2% low-melt agarose gel and gel-purified with PureLink Quick Gel Extraction Kit (LifeTechnologies). Cloning was performed with the Topo TA Cloning Kit for Subcloning with either chemically competent or electrocompetent bacteria (LifeTechnologies) according to the manufacturer’s instructions. Miniprep and DNA-Sequencing of at least three clones was performed by Microsynth AG (Balgach, Switzerland). Sequencing results were analyzed with the BiQAnalyzer software.^38^

#### Statistical analysis

Data were log transformed to achieve normal distribution, paired *t*-tests were calculated on mRNA expression data for tumor samples and respective adjacent mucosa samples from the same patient. Number of patients and two-tailed significance levels are stated. The nonparametric Spearman Correlation was calculated without log transformation and the Spearman Correlation Coefficient and the two-tailed significance levels are given. All calculations were performed with SPSS Version 18, graphs were drawn using GraphPad Prism Version 5.

### Results

#### Genes that control the metabolism of calcidiol are deregulated in colorectal tumor samples

**Figure 1:**
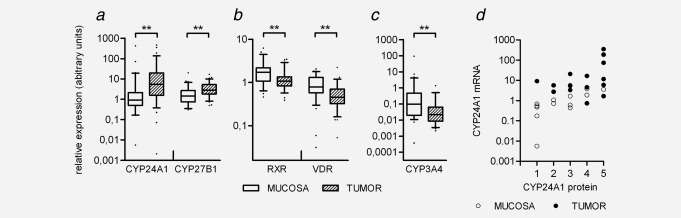
mRNA expression of vitamin D pathway genes differs between colorectal tumors and respective adjacent mucosa. mRNA expression was assessed in colorectal tumors and adjacent mucosa by qRT-PCR. 2-tailed paired *t*-tests on log transformed data were computed (*p < 0.05, **p < 0.001). Relative expression of CYP24A1 (*n* = 69) and CYP27B1 (*n* = 68) are shown in (*a*), RXR (*n* = 63) and VDR (*n* = 68) in (*b*), CYP3A4 (*n* = 70) in (*c*). Median, interquartile range and whiskers representing 5th to 95th percentile are shown. CYP24A1 protein expression in tumors and adjacent mucosa samples was determined by immunofluorescence stainings and was graded from 1–5 (low-high, *x*-axis), respective mRNA expression of CYP24A1 is shown on the *y*-axis (*d*).

Expression of CYP24A1 was significantly higher in colorectal tumor tissue compared with the respective adjacent mucosa (4.8-fold change; *n* = 69; *p* < 0.001; Table1 and Fig. 1*a*). This overexpression was confirmed on protein level by immunofluorescence staining of paraffin sections (Supporting Information Fig. S1). In the samples analyzed, CYP24A1 mRNA expression and protein expression strongly correlated (*n* = 29, Spearman Correlation Coefficient SCC = 0.615, *p* < 0.001, Fig. 1*d*). CYP24A1 overexpression did not correlate with any of the pathophysiological characteristics shown in Table1, such as lymph node involvement or tumor location. As CYP24A1 transcription is highly induced by 1,25-D_3_, we also determined expression of the 1,25-D_3_ synthesizing enzyme CYP27B1, as well as of VDR and RXR, the transcription factors that mediate 1,25-D_3_-dependent CYP24A1 transcription. CYP27B1 mRNA was significantly upregulated in the tumor tissue compared with the adjacent normal mucosa (twofold change; *p* < 0.001; Fig. 1*a*), whereas VDR and RXR were both significantly downregulated (*p* < 0.001, Fig. 1*b*). Specificity protein 1 (SP1) involved in both basal and vitamin D-dependent transcription of CYP24A1 was upregulated in adenocarcinomas suggestive of an 1,25-D_3_ independent upregulation (*p* < 0.001, Supporting Information Fig. S2). As an indicator of local 1,25-D_3_ activity, we measured the expression of one of its target genes CYP3A4. CYP3A4 mRNA expression was strongly decreased in colorectal tumor tissue compared with the adjacent normal mucosa (*p* < 0.001, Fig. 1*c*) consistent with the hypothesis that 1,25-D_3_ activity is suppressed in the context of CYP24A1 overexpression.

**Table 1:** Patient cohort

Tissue	Copy number analysis	mRNA expression	Overlap
*n*	127	69	59
Gender			
Female	66 (52%)	34 (49%)	31 (53%)
Male	61 (48%)	34 (49%)	28 (47%)
Unknown	0	1	0
Age			
Mean ± SD	69.5 ± 12.6	68.8 ± 11.1	68.7 ± 8.0
Tumor grading			
Grade 1	1	1	1
Grade 2	106	53	46
Grade 3	18	12	10
Unknown	2	3	2
Lymph node infiltration			
0	58	30	25
1	30	17	15
2	35	17	15
3	1	1	1
Unknown	3	4	3
Site of the primary tumor			
Cecum/ascending/transverse colon	48	26	23
Descending/sigmoid colon	46	24	24
Rectum	30	17	11
Unknown	3	2	1

Thus, CYP24A1 overexpression does not correlate with VDR or RXR expression. Since both VDR and RXR were significantly downregulated in the tumors, it is unlikely that the physiological 1,25-D_3_-VDR-induced transcription accounts for CYP24A1 overexpression.

#### CYP24A1 promoter methylation is comparable between tumor and normal mucosa

**Figure 2:**
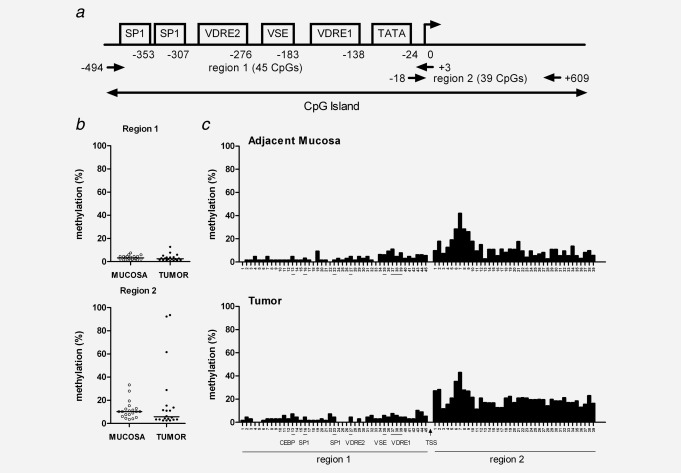
Promoter methylation of CYP24A1 in adjacent mucosa and tumor tissue. Schematic overview of the CYP24A1 promoter region including position of regulatory elements (vitamin D responsive elements (VDRE1 and VDRE2), specificity protein 1 (SP1), vitamin D stimulatory element (VSE) and TATA box) as well as position of bisulfite genomic sequencing primers (black arrows) are shown (*a*). Bisulfite genomic sequencing of the *CYP24A1* promoter was performed in 20 human adenocarcinoma and 20 adjacent mucosa samples. For each region, the mean methylation per sample is blotted, median is indicated (*b*). Incidence of CpG nucleotide methylation is shown for tumor and adjacent mucosa (*c*).

To test the hypothesis that the expression of CYP24A1 is low due to dense promoter methylation in normal colon and that hypomethylation upregulates CYP24A1 during carcinogenesis, we determined the methylation status of two distinct regions of the *CYP24A1* promoter (Fig. 2*a*) in samples of colorectal tumors and their respective adjacent mucosa (*n* = 20). We found very low levels of methylation (<3% mean methylation) in promoter region 1 in both adenocarcinomas and the adjacent normal tissue, indicating the absence of methylation-dependent transcriptional silencing (Fig. 2*b*). A similar low level of methylation (<4%) was observed in the tissue obtained from a healthy control subject (data not shown). Interestingly, methylation of promoter region 2 was higher than that in region 1 but, as for promoter region 1, there was no significant difference between the levels of methylation in tumor tissue and adjacent mucosal tissue as calculated by paired *t*-tests on log-transformed data, respectively (median region 1: mucosa: 4%, tumor 2%; region 2: mucosa 10%, tumor 7%). In addition, promoter methylation of *CYP24A1* did not correlate with mRNA expression (Supporting Information Table S1). Figure 2*c* shows the average CpG methylation frequency in tumor and mucosa. The transcription start site (TSS) and TATA box are located between region 1 and 2 and do not contain CpGs. There appeared to be no difference in the methylation patterns of responsive elements between tumor and mucosa. A methylation hot spot was observed in region 2 approximately 220bp downstream of the TSS (Fig. 2*c*). In summary, the similar methylation levels found in adjacent mucosa and tumor samples for both *CYP24A1* promoter regions 1 and 2 do not suggest involvement of DNA methylation in regulation of CYP24A1 expression.

#### 60% of colorectal tumors carry increased number of copies of the *CYP24A1* gene

**Figure 3:**
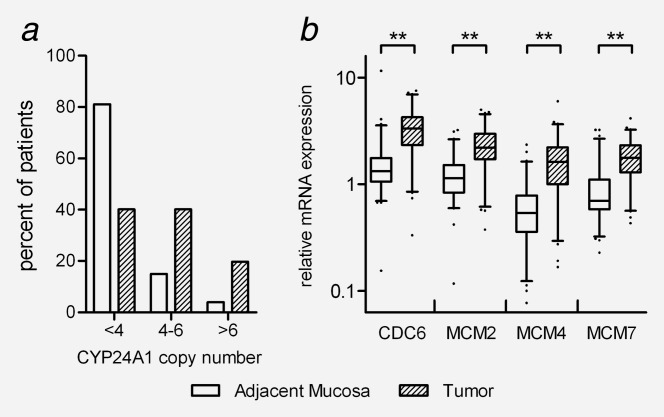
CYP24A1 gene copy-number and proliferation marker expression in colorectal tumors. Genomic copy-number of *CYP24A1* was determined in tumor tissue and adjacent mucosa (*n* = 127). Patients were grouped in three categories, less than four copies (normal), four to six copies (amplified) and more than six copies (highly amplified), data are presented as % of total patients (*a*). mRNA expression of the proliferation markers CDC6, MCM2, MCM4 and MCM7 were assessed in tumor and adjacent mucosa. Paired samples *t*-test was computed on log transformed data, 2-tailed *p*-values (** *p* < 0.001) are indicated (*b*). Median, interquartile range and whiskers representing 5th to 95th percentile are shown.

The long arm of chromosome 20 and specifically the region 20q13 are amplified in various cancers, including colorectal cancer. To assess the genomic copy-number of *CYP24A1*, located at 20q13.2, we developed a quantitative real-time PCR assay based on the methods of Ponchel *et al*.^34^ and Muhamad *et al*.^39^ We determined *CYP24A1* copy-number in colorectal tumors and respective adjacent mucosa of 127 patients (Table1). As the assay does not give absolute values, we considered fewer than four copies as normal, four to six copies of *CYP24A1* as moderately amplified, and greater than six copies of *CYP24A1* as highly amplified. Of the adjacent normal mucosa samples studied, 103 (81%) exhibited normal *CYP24A1* copy-numbers and 24 (19%) samples carried more than four copies of *CYP24A1* (Fig. 3*a*). However, of the adenocarcinoma samples studied, only 51 (40%) exhibited normal *CYP24A1* copy-number and 76 (60%) had elevated copy-number. Of these 76 adenocarcinomas, 51 (67%) had four to six copies and 25 (33%) had more than six copies. Figure 3*a* shows the distribution of patients in the above mentioned groups. *CYP24A1* copy-number did not correlate with patient age, sex, or anatomical location of the tumor. To validate our results of the PCR assay, we performed fluorescence *in situ* hybridization (FISH) analysis on two cell lines and five patients. The FISH analysis of the melanoma cell line VM-17 used as a positive control in our qPCR assay, showed clear CYP24A1 gene amplification. At the same time in the colon cancer cell line Caco-2/15 we found both *CYP24A1* gene amplification as well as chromosome 20 polysomy. The FISH analysis of the patient samples also confirmed increases in copy-numbers, however, it suggested that the higher copy-numbers are primarily due to chromosome 20 polysomy and only partially to 20q13.2 gene locus amplification.

#### High *CYP24A1* gene copy-number correlates with increased mRNA expression

To determine the impact of *CYP24A1* copy-number gain on mRNA expression, we performed correlation analysis between number of *CYP24A1* copies and mRNA expression in the same patient cohort (*n* = 58, Table1). We used the nonparametric Spearman Correlation Coefficient to test for statistical dependence between CYP24A1 mRNA expression and *CYP24A1* copy-numbers. A Spearman Correlation Coefficient (SCC) of “1” indicates almost linear correlation, “0” absence of a correlation. We found a significant correlation between copy-number status and mRNA expression in the overall patient cohort (SCC = 0.379). However, in the subgroup of patients with high *CYP24A1* copy-number (6–11), the correlation increased considerably (SCC = 0.580; Table2, dot-blots shown in Supporting Information Fig. S3) indicating that high copy-numbers are associated with increased mRNA expression, consistent with the idea that copy-number gain results in enhanced *CYP24A1* transcription.

**Table 2:** CYP24A1 gene copy-number correlates with increased mRNA expression

		Correlation CYP24A1 copy number—mRNA	
CYP24A1 copy number	*n*	SCC	Sig. (two-tailed)
Total	118	0.379	0.001
>6	13	0.580	0.038

#### CYP24A1 mRNA expression correlates with proliferation

Increased expression of CYP24A1 lowers the half-life of 1,25-D_3_ and thus suppresses local 1,25-D_3_ concentrations. Such an outcome is predicted to promote proliferation and impair differentiation. As more than 75% of tumors were classified as moderately differentiated (*i.e*., grade 2; Table1), we were unable to assess the relationship between tumor grade and CYP24A1 expression. Therefore, to assess the possible impact of CYP24A1 mRNA expression on the proliferative potential of the tumors, we quantified mRNA expression of several genes responsible for initiating cell cycle turnover, including cell division cycle 6 homolog (CDC6) and mini-chromosome maintenance complex components 2, 4 and 7 (MCM2, MCM4, MCM7). All licensing factors were expressed at elevated levels in the colorectal tumor samples compared with the adjacent mucosa (log transformed data; *p* < 0.001; Fig. 3*b*). Furthermore, CYP24A1 mRNA expression correlated with CDC6 (SCC = 0.596), MCM2 (SCC = 0.566), MCM4 (SCC = 0.569) and MCM7 (SCC = 0.568) mRNA expression in the overall cohort (Table3, Supporting Information Fig. S4). Interestingly, in the subgroup of patient samples with more than six copies of CYP24A1, these correlations were significantly strengthened (CDC6: SCC = 0.607; MCM2: SCC = 0.829; MCM4: SCC = 0.789; MCM7: SCC = 0.768, Table3, Supporting Information Fig. S5). Therefore, high *CYP24A1* copy-number was associated with upregulated expression of key activators of cell cycle turnover raising the possibility that enhanced 1,25-D_3_ degradation and suppression of local 1,25-D_3_ levels removes an important inhibitory restraint on cell proliferation.

**Table 3:** CYP24A1 mRNA expression correlates with expression of markers of proliferation

				>6 genomic copies of CYP24A1		
Gene	*n*	SCC	Sig. (two-tailed)	*n*	SCC	Sig. (two-tailed)
CDC6	120	0.596	0.001	15	0.607	0.016
MCM2	120	0.566	0.001	15	0.829	0.001
MCM4	120	0.569	0.001	15	0.789	0.001
MCM7	120	0.568	0.001	15	0.768	0.001

### Discussion

In this study, we show that high *CYP24A1* copy-number leads to increased mRNA expression of CYP24A1. Furthermore, CYP24A1 expression correlated with several proliferation markers, suggesting that high CYP24A1 expression might result in stronger proliferating tumors. In addition, we provide evidence that overexpression of CYP24A1 in colorectal cancer is not caused by promoter hypomethylation.

Changes in 1,25-D_3_ levels are dependent on the relative expressions of the 1,25-D_3_ synthesizing enzyme CYP27B1 and the degrading enzyme CYP24A1. Although CYP24A1 is overexpressed in most cancer types,^9^,^40^–^43^ reports on changes in CYP27B1 expression are inconsistent.^44^ In our colorectal cancer patient cohort the expression of mRNAs encoding both enzymes was elevated. Previously, coordinate upregulation of CYP24A1 and CYP27B1 was observed in breast cancer with an apparent net outcome of enhanced 1,25-D_3_ catabolism.^41^ As both, the absolute expression levels as well as the fold-increases of CYP24A1 were higher than for CYP27B1 in tumor samples, the results suggest a shift to enhanced 1,25-D_3_ breakdown in our patient cohort. Consistent with a local reduction in 1,25-D_3_ levels, we observed reduced expression of the 1,25-D_3_ target gene CYP3A4 in the tumor samples. Comparing CYP3A4 expression levels between tumor and normal mucosa in the same patient controls for impact of possible drug treatment on CYP3A4 level. Our study is the first to show that in colorectal tumors not only VDR^9^,^10^ but also RXR, two key mediators of 1,25-D_3_-dependent transcription of CYP24A1 were both downregulated, while SP1 was elevated in the tumors. This suggests an 1,25-D_3_-independent upregulation of CYP24A1, possibly via a noncanonical control mechanism such as promoter hypomethylation or increased CYP24A1 copy-number.

As the methylation status of *CYP24A1* in normal colon and colonic tumor tissue has been previously unknown, we hypothesized that CYP24A1 expression may be limited by high levels of promoter methylation in normal colon as observed in placenta and that hypomethylation during carcinogenesis may drive strong increases in transcription. However, we observed only low levels of *CYP24A1* promoter methylation both in a sample of healthy colon and in the apparently normal mucosa adjacent to tumors in patients with colorectal cancer. Previously, we have demonstrated that in colon cancer cell lines the methylation status of the CGI of the *CYP24A1* promoter was not involved in regulation of CYP24A1 expression.^45^ Taken together, our results indicate that hypomethylation of the *CYP24A1* promoter is not responsible for the upregulation of CYP24A1 expression in colorectal tumors.

During carcinogenesis, genomic rearrangements and chromosome aneuploidy are caused by increased genomic instability. Comparative genome hybridization (CGH) studies have demonstrated that there is a nonrandom distribution of copy-number gains in colorectal cancer, with a hotspot on chromosome 20q^12^,^28^,^32^. Although the frequency and level of 20q13 gain corresponding to the location of *CYP24A1* and neighboring genes varied significantly in colorectal tumors^31^,^33^, this suggests a selection advantage for tumors carrying these amplifications. Recently, it was suggested that this advantage is not caused by a single gene but rather a cluster of genes, including aurora kinase A (*AURKA*, 20q13.2), which is involved in mitotic spindle assembly and chromosome segregation. In a recent study, gene amplification of *AURKA* was linked to increased expression of AURKA mRNA^29^, however, no increase in CYP24A1 expression was observed. In this study, we found that enhanced CYP24A1 expression correlated with gene copy-number and this was more significant if the CYP24A1 gene copy-number was greater than 6. One of the transcription factors that drives basal transcription (SP1) was overexpressed in colorectal tumors. This raises the possibility that high copy-number together with increased basal transcription drives CYP24A1 overexpression.

The copy-number gains detected in 19% of adjacent mucosa samples suggest that, as reported previously, this tissue cannot be considered healthy normal tissue as it already harbors alterations^46^,^47^. Inclusion of a healthy control group in future studies would assist the evaluation of adjacent tissue for genetic and phenotypic abnormalities. The approach taken in the present study to compare samples of tumor tissue with the adjacent mucosa from the same patient has yielded clear evidence for increased number of *CYP24A1* copies in tumors and has the advantage of controlling for differences in nutrition, lifestyle, environment, and genetic predisposition.

The high frequency of *CYP24A1* copy-number gains (60% of all tumor samples) in our patient cohort suggested a selection advantage for tumors carrying this modification. Therefore, it is of great importance to determine the consequences of *CYP24A1* copy-number gain. Increased CYP24A1 activity limits the biological half-life of 1,25-D_3_ and thereby counteracts the anti-tumorigenic effects of 1,25-D3, possibly resulting in increased proliferation. Previously, we demonstrated a correlation between CYP24A1 protein expression and the expression of the proliferation marker Ki-67 protein in colorectal tumors^9^. In this study, we examined whether CYP24A1 mRNA expression correlates with very early phases of DNA replication, namely with expression of replication licensing factors. CDC6 is involved in the loading of the MCM helicase complex onto DNA. MCM2 expression was shown previously to correlate with the proliferative potential of colorectal cancers and MCM2 overexpression was also detected in gastric carcinomas^48^,^49^. We found significant correlation between CYP24A1 expression and the expression of replication licensing factors. Interestingly, the correlation was considerably strengthened in the high copy-number group, expressing extremely high levels of CYP24A1 protein, suggesting that colorectal tumors harboring more than six copies of CYP24A1 are highly proliferative. Whether CYP24A1 overexpression is sufficient to drive tumor growth is not yet clear. However, we previously showed that in cells with high basal CYP24A1 expression, the anti-proliferative effects of 1,25-D_3_ can only be restored by inhibition of CYP24A1 activity^50^.

In conclusion, upregulation of CYP24A1 gene expression in colorectal cancer is not caused by DNA hypomethylation. Instead, we found high *CYP24A1* gene copy-number in 60% of colorectal tumors and the copy-number gain was independent of sex, anatomical location and age. Furthermore, high *CYP24A1* copy-number correlated with increased mRNA and protein expression, suggesting that increased *CYP24A1* copy-number contributes to the overexpression of CYP24A1. In addition, CYP24A1 expression correlated with recognized proliferation markers including CDC6, MCM2, MCM4 and MCM7. Indeed, the correlation was strongest in samples from patients with more than six genomic copies of *CYP24A1*. Assuming that 24-hydroxylase activity changes in concert with CYP24A1 mRNA and protein expression it seems reasonable to hypothesize that the role of CYP24A1 in malignant transformation arises from downregulation of 1,25-D_3_ leading to loss of its anti-proliferative, pro-differentiating effects. Consistent with this idea, CYP3A4 a gene whose expression is positively modulated by 1,25-D_3_, was downregulated in colorectal tumor samples when compared with adjacent mucosa tissue. Mechanistic studies are required to prove causality of the observed relationship between CYP24A1 overexpression and proliferation. If overexpression of CYP24A1 directly affects tumor proliferation, tumor-targeted treatment with CYP24A1-specific inhibitors may be effective in slowing tumor growth.

## Abbreviations

1,25-D3, calcitriol: 1,25-dihydroxyvitamin D3
AURKA: aurora kinase A
CDC6: cell division cycle 6 homolog
CGH: comparative genome hybridization
CGIs: CpG islands
CRC: colorectal cancer
CYP24A1: 1,25-dihydroxyvitamin D 24-hydroxylase
CYP27B1: 25-hydroxyvitamin D3 1α-hydroxylase
MCM: mini-chromosome maintenance complex
RXR: retinoid X receptor
SCC: spearman correlation coefficient
SP1: specificity protein 1
TSS: transcription start site
VDR: vitamin D receptor
FISH: fluorescence *in situ* hybridization
