# Supplementary figures and images for: Increased copy-number and not DNA hypomethylation causes overexpression of the candidate proto-oncogene CYP24A1 in colorectal cancer
Source: Int J Cancer. 2013 Apr 5;133(6):1380–8. doi: 10.1002/ijc.28143 (PMC3807607; doi:10.1002/ijc.28143)

**Supplementary Figure 1**

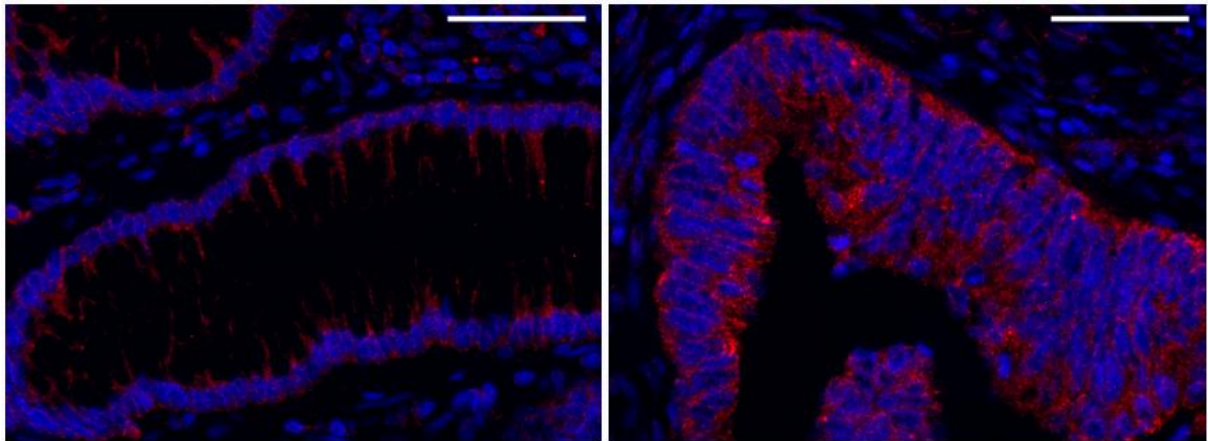

Supplement: Supplementary file 1 [file ijc0133-1380-sd1.docx]

**Supplementary Figure 2**

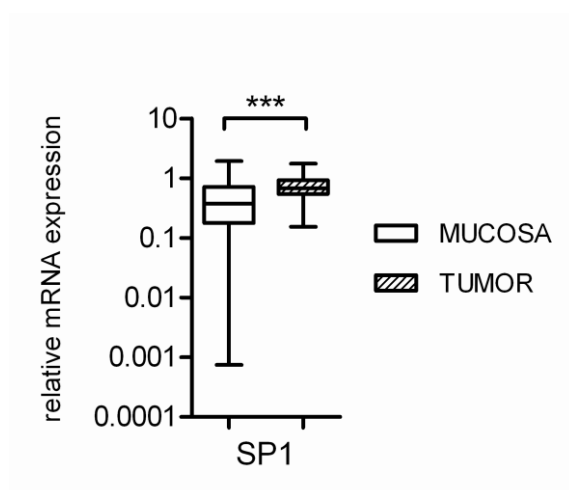

Supplement: Supplementary file 2 [file ijc0133-1380-sd2.pdf]

Supplementary Figure 3

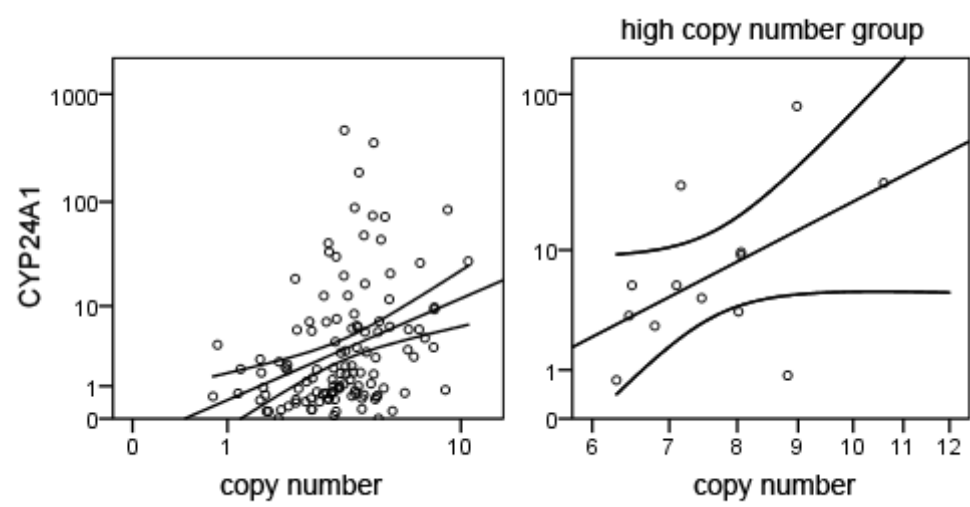

Supplement: Supplementary file 3 [file ijc0133-1380-sd3.pdf]

Supplementary Figure 4

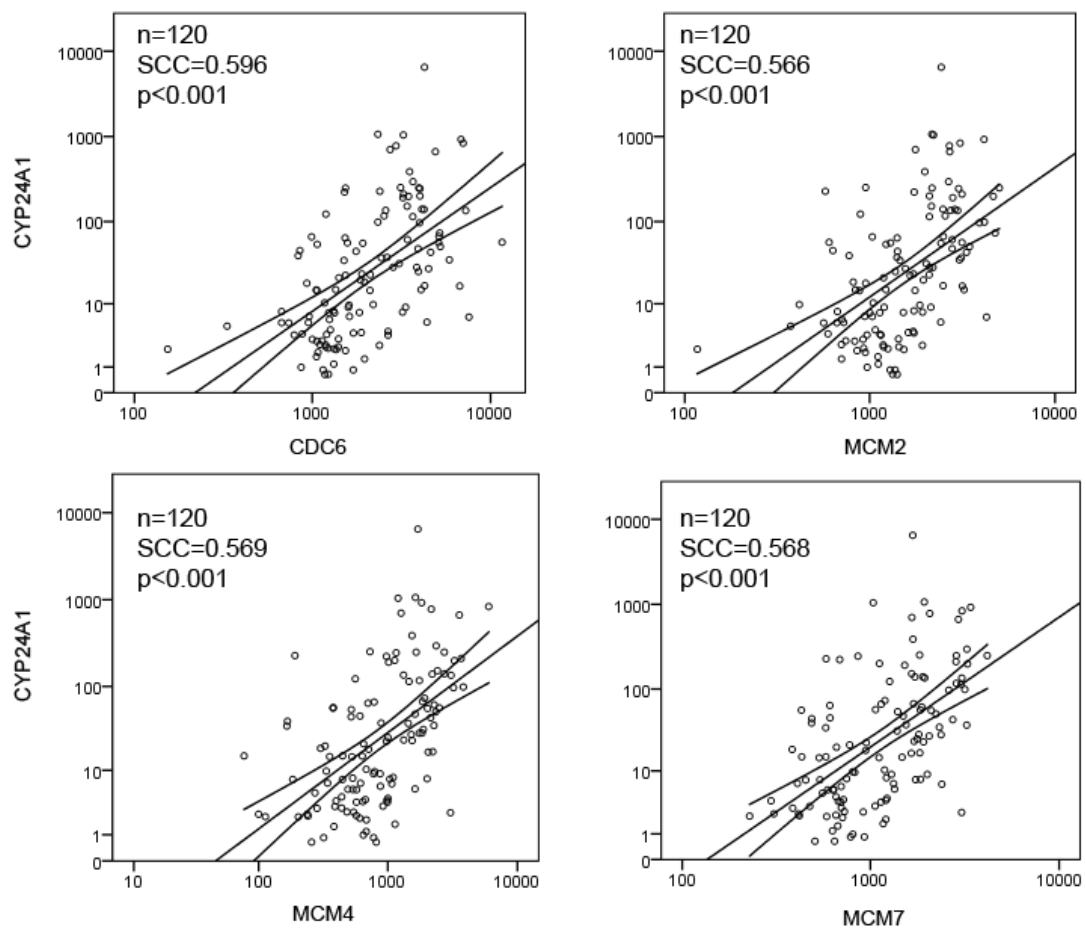

Supplement: Supplementary file 4 [file ijc0133-1380-sd4.pdf]

## Supplementary Figure 5

high copy number group

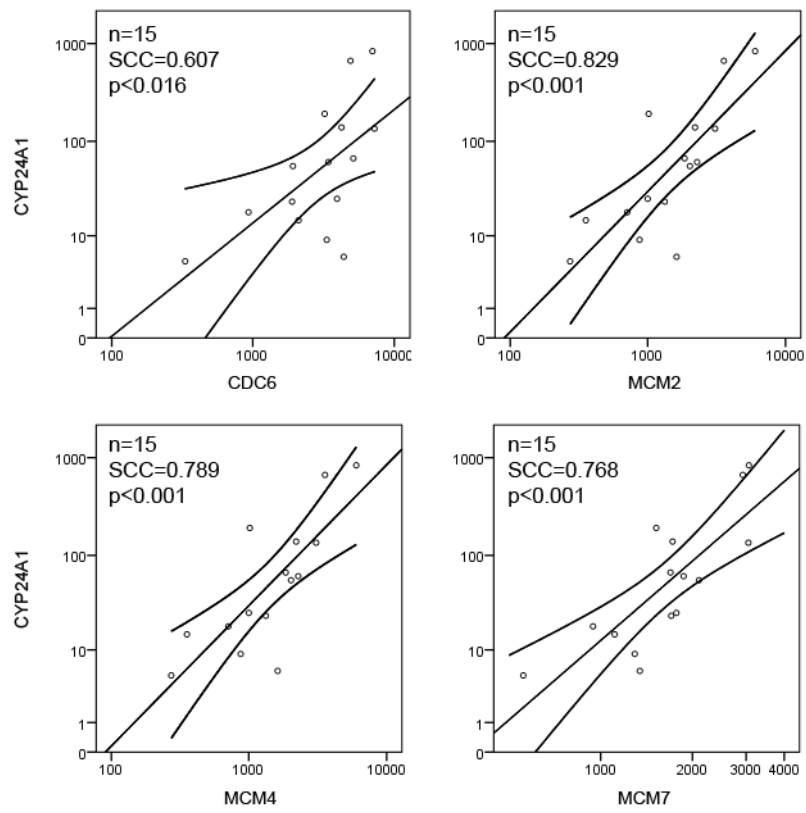

Supplement: Supplementary file 5 [file ijc0133-1380-sd5.pdf]
