# Supplementary material for: Increased copy-number and not DNA hypomethylation causes overexpression of the candidate proto-oncogene CYP24A1 in colorectal cancer
Source: Int J Cancer. 2013 Apr 5;133(6):1380–8. doi: 10.1002/ijc.28143 (PMC3807607; doi:10.1002/ijc.28143)
Supplement: Supplementary file 6 [file ijc0133-1380-sd6.pdf]

**Supplementary Table 1. Detailed overview of CYP24A1 promoter methylation in region 1 and 2 and CYP24A1 mRNA expression in tumors relative to mucosa**

|  | CYP24A1 | **MUCOSA** | | TUMOR | |
| --- | --- | --- | --- | --- | --- |
|  | mRNA tumor | % Methylation | | % Methylation | |
| Patient | relative to mucosa | Region 1 | Region 2 | Region 1 | Region 2 |
| 1 | (0,04 fold) ↓ | 3 | 15 | 13 | 92 |
| 2 | ↑ (93 fold) | 4 | 8 | 2 | 3 |
| 3 | ↑ (1 fold) | 2 | 13 | 1 | 14 |
| 4 | ↑ (1 fold) | 2 | 11 | 1 | 29 |
| 5 | ↑ (5 fold) | 8 | 33 | 3 | 4 |
| 6 | ↑ (50 fold) | 2 | 28 | 6 | 12 |
| 7 | ↑ (19 fold) | 4 | 10 | 2 | 15 |
| 8 | (0,04 fold) ↓ | 4 | 4 | 5 | 5 |
| 9 | ↑ (2 fold) | 4 | 10 | 1 | 62 |
| 10 | (0,31 fold) ↓ | 4 | 12 | 8 | 94 |
| 11 | n.d. | 1 | 10 | 4 | 6 |
| 12 | ↑ (9 fold) | 4 | 5 | 4 | 3 |
| 13 | ↑ (3 fold) | 3 | 10 | 1 | 11 |
| 14 | (0,02 fold) ↓ | 5 | 6 | 3 | 4 |
| 15 | ↑ (17 fold) | 2 | 3 | 2 | 11 |
| 16 | ↑ (89 fold) | 6 | 12 | 2 | 3 |
| 17 | n.d. | 1 | 9 | 3 | 3 |
| 18 | ↑ (8 fold) | 1 | 4 | 4 | 3 |
| 19 | ↑ (27 fold) | 1 | 19 | 1 | 3 |
| 20 | ↑ (119 fold) | 6 | 8 | 1 | 3 |
